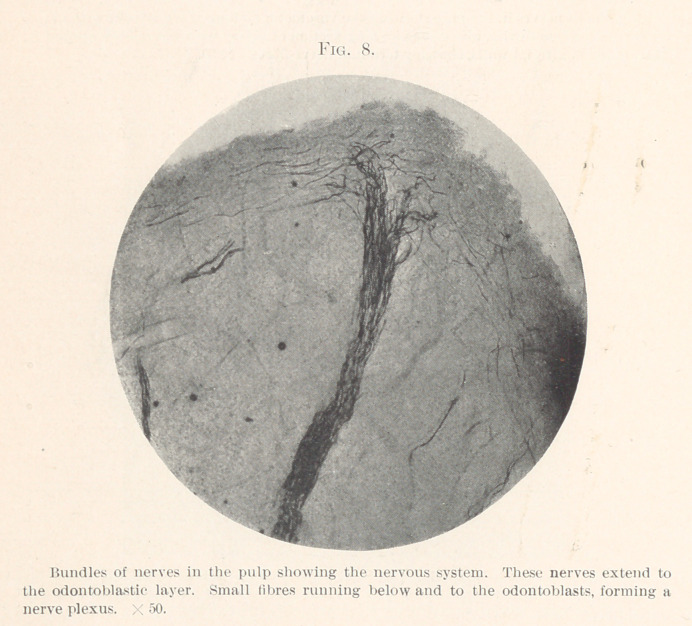# The Vasomotor System of the Pulp

**Published:** 1904-02

**Authors:** Eugene S. Talbot

**Affiliations:** Chicago


### International Dental Journal.

Vol. XXV. February, 1904. No. 2.

### Original Communications.[1]

### THE VASOMOTOR SYSTEM OF THE PULP.[2]

BY EUGENE S. TALBOT, M.D., D.D.S., CHICAGO.

In 1733 Stephen Hales[3] first published the idea that small arteries changed their caliber. He devised the following ingenious experiment: Tying a brass tube into the aorta of a dog and employing a head pressure equal to the normal aortic tension, he injected water and measured the outflow per minute from the divided vessels of the intestines. He found cold water diminished, while hot water increased the flow. He also showed (by the action of drugs) that one set of agents contracted the vessels and lessened the outflow, while another set widened the vessels and increased the flow.[4]

The chief dominating centre of the non-striped muscles of the arterial system with motor nerves (vasomotor, vasoconstrictor,

[1] The editor and publishers are not responsible for the views of authors of papers published in this department, nor for any claim to novelty, or otherwise, that may be made by them. No papers will be received for this department that have appeared in any other journal published in the country.

[2] Read before the American Medical Association, Section on Stomatology, at New Orleans, La., May 5 to 8, 1903.

[3] Statical Essays, 1733, vol. xi.

[4] A history of some of the experiments from this period to the present time may be found in Text-Book of Physiology, by E. A. Schäfer.

vasohypertonic) lies in the medulla oblongata. The nerves which pass to the blood-vessels are known as the vasomotor nerves. Without mentioning the experiments which have been made, I might say, in a general way, stimulation of this nerve-centre causes contraction of all the arteries, resulting in great increase of the arterial blood-pressure and swelling of the veins of the heart. Paralysis of this centre causes relaxation and dilatation of all the arteries, resulting in an enormous fall of the blood pressure.[1]

The sympathetic nerves consist of two chains of ganglia, one on each side of the spinal cord. Their function is to stimulate the viscera, glands, heart, blood-vessels, and unstriped muscles of the body generally.

There are four small ganglia [2] connected with the fifth nerve. The ophthalmic, spheno-palatine, otic, and submaxillary. Each has three roots derived from motor, sensory, and sympathetic nerves respectively, and varying members of branches of distribution. These cells are multipolar, thus resembling the cells of sympathetic ganglia and differing from those of the ganglia of the posterior spinal nerve-roots and Gasserian ganglion.

Each ganglion is a reddish-gray color, soft in consistence but enclosed in a strong fibrous sheath. It is connected above and below by an ascending and a descending trunk, and with at least one spinal nerve by one or two communicating branches. Each ganglion distributes different branches, which either directly or through the intervention of a secondary plexus supply blood-vessels.

The second or superior maxillary division of the fifth pair of nerves gives off the posterior superior, middle superior, and anterior superior dental nerves. These three nerves form a plexus and loops of filament which pass to the tips of the roots to form the dental pulp. The third division of the fifth, the inferior maxillary, a mixed sensory and motor nerve, enters the inferior dental canal, passing forward towards the symphysis menti, supplying branches to the teeth and gums.

The Gasserian ganglion received filaments from the carotid plexus of the sympathetic. Connected with the fifth are the four small ganglia already mentioned, which form the whole of the cephalic portion of the sympathetic. With the first division is

---

[1] Landois and Stirling, Human Physiology.

[2] Gerrish Anatomy.

connected the ophthalmic ganglion, with the second, the submaxillary ganglion. All the four receive sensitive filaments from the fifth, motor and sympathetic filaments from various sources. The ganglia are also connected with each other and the cervical portion of the sympathetic.

According to the "American Text-Book of Physiology," the vasomotor apparatus consists of three classes of nerve-cells.[1] The cell bodies of the first class lie in sympathetic ganglia, their neuraxons passing directly to the smooth muscles in the wall of the vessels; the second are situated at different levels in the cerebrospinal axis, their neuraxons passing thence to the sympathetic ganglia by way of the spinal and cranial nerves; the third are placed in the bulb and control the second through interspinal and intercranial paths. The nerve-cells of the first class lie wholly without the cerebrospinal axis, the third wholly within, while the second is partly within, partly without, and binds the remaining two together.

The vasomotor fibres for the face and mouth have been found in the cervical sympathetic by Dastre and Morat, leaving the cord[2] in the second and fifth dental nerves and uniting (at least for the most part) with the trigeminus by passing, according to Morat,[3] from the superior cervical sympathetic ganglion to the ganglion of Gasser, and thence to the fifth nerve. The nerves of the cerebrospinal system, with the exception of the olfactory, are medullated nerves.

The vasomotor nerves are axis cylinder processes of the sympathetic ganglion cells. They follow for a time the course of the corresponding spinal nerves. Intermingled with the medullated fibres are always found gray or non-medullated fibres. According to Shäfer,[4] these fibres frequently branch; the medullated fibres rarely do, except near their termination. The sympathetic nerves are largely made up of these fibres as they approach their peripheral distribution, and possess a thin medullary sheath.

From what has been said it will be seen that the intimate rela-

---

[1] By "nerve-cells" is meant the cell body with all its processes,—namely the neuraxons, or axis cylinder processes, and dentrites, or protoplasma processes.

[2] Dastre and Morat, 1884, pp. 116, 120.

[3] Morat, 1889, p. 201.

[4] Essentials of Histology, p. 118.

tion of the fifth nerve is a motor nerve in mastication and a sensory nerve to the great surface, both external and internal, which belongs to the face and the anterior part of the cranium. From the great size and the large portion of the medulla with which it is connected, there can be no question but that its sympathetic and vasomotor connection is established.

The nerve-trunks as they pass through the lower jaw are made up of nerve-fibres gathered together into bundles or funiculi, held together by connective tissue and called the perineurium. The connective tissue which unites a number of the bundles of funiculi is called epineurium. The cut ends under the microscope resemble very much the end of an ocean cable, the wires representing the nerve-fibre, and the rubber covering the connective tissue sheath.

From this nerve-trunk, smaller medullated nerve-fibres are given off at the nodes of Ranvier, which pass up and into the apical foramina of the roots of the teeth. Sometimes there are two and again three or ten to twenty-five nerve-fibres entering the foramina. The number depends upon the size of the opening. In my paper last year on "The Evolution of the Pulp," I demonstrated that in animals whose teeth were in continuous eruption during life, the pulp was larger at the opening than in the pulp-chamber. In ascending to man, the second teeth have small openings, especially later in life. In the very nature of things, as the root calcifies, especially in exostosis, the openings grow smaller. The number of nerves entering the foramina, then, will depend upon the size of the apical opening.

In a general way the motor, sensory, and the sympathetic nerves have been traced from their source to the roots of the upper and lower teeth. Text-books demonstrate the peripheral end organs to other structures of the body. In no case (to my knowledge) has the character of the nerves of the pulp been demonstrated.

At the meeting of this Section last year, Dr. Vida A. Latham, in a paper entitled "Résumé of the Histology of the Dental Pulp," showed the nerve supply to the pulp in different forms and also one illustration of the vasomotor system of the pulp. Part of our special work in the laboratory this year has been to demonstrate the character of the nerves of the pulp.

Pulp study has been conducted by Dr. Latham and myself in different laboratories for the past four years. For this work, teeth have been collected to the number of over four thousand. These have been cracked open and over two thousand specimens of pulps have been placed in different fluids ready for cutting, staining, and mounting for the microscope. All the different methods of preparation of nerve-tissue have been used as reported by Dr. Anderson in a paper entitled " Notes on Pulp Technique."

The nerve-fibres, after leaving the main trunk in the jaw, evidently enter the apical foramina in single nerve bundles. In many cases these nerve bundles continue the entire length of the pulp without branching. On the other hand, the branching in many cases begins after the trunk nerves have passed through the apical foramina. In Fig. 1, when the tooth was extracted the pulp protruded from the end of the root, the opening being quite large. In this illustration the nerve-fibres are shown from the inferior dental nerve extending through the apical foramina in the root-canal of the tooth. These seem to run in a bundle or funiculi, with the exception of one fibre, which is isolated at the root. Fig. 2 shows bundles of nerve-fibres loosely arranged running in different directions. Between these bundles may be seen many single nerve-fibres running in all directions. In the centre of the field is an artery cut crosswise with terminal fibres encircling it two-thirds around. Fig. 3 beautifully illustrates the vasomotor nerves in their relation to the blood-vessels. The blood-vessels and nerves run in the same direction. In the centre of the field may be seen four arteries. Nerve-fibres are notably running the entire length between, but they cross and recross at different localities. Nerve-fibres in bundles and singly cover the entire field. Fig. 4 shows bundles of fibres, with many single fibres throughout the field. In the centre may be seen an artery cut lengthwise branching in two directions. The most interesting of all, however, is an artery cut crosswise with vasomotor terminal nerves encircling it. Fig. 5 demonstrates the vasomotor system more thoroughly. In the centre of the field may be seen nine arteries cut lengthwise and one cut crosswise. Bundles of nerve-fibres run between the arteries and along the arterial walls. Nerve-fibres are seen crossing and recrossing the arterial walls, sometimes in bundles and again in single terminal fibres. In the cross-cut artery a nerve-fibre may be seen almost encircling it. Fig. 6 shows an enlarged artery cut lengthwise, while just below it may be seen an artery running towards it at right angles. In this artery only the outer surface is seen. In both arterial coats terminal nerve-fibres are well shown. Fig. 7 shows the ends of the nerve cut crosswise. An artery may also be seen with a nerve encircling it. Fig. 8 illustrates the crown end of the pulp with a bundle of nerve-fibres which have extended intact the entire length of the pulp and is distributing its fibres throughout the odontoblastic layer.

In consideration that the blood-vessels and nerves pass through the pulp in a wavy direction and not in straight lines, to have been able to obtain so many beautiful specimens showing so clearly and distinctly the vasomotor system is fortunate.

I am obliged to Dr. Ludwig Hektoen for verifying these illustrations, and to Dr. Martha Anderson for valuable services.

## Figures and Tables

**Fig. 1. f1:**
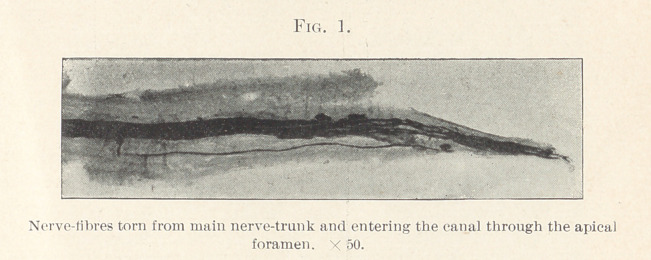


**Fig. 2. f2:**
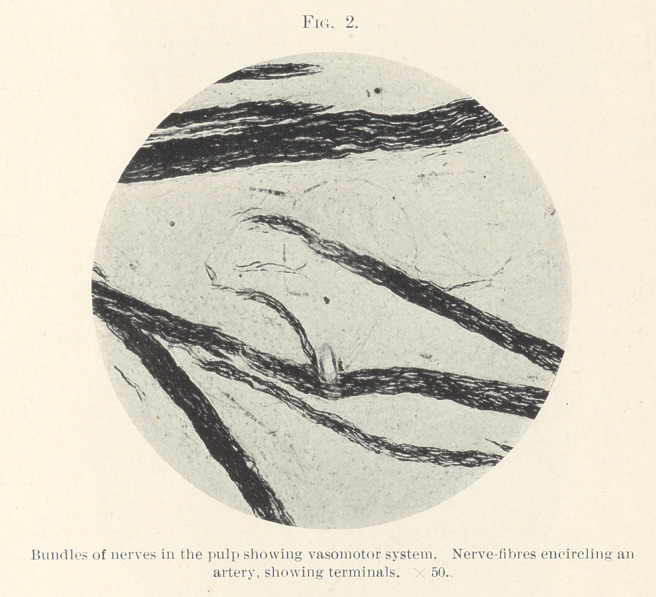


**Fig. 3. f3:**
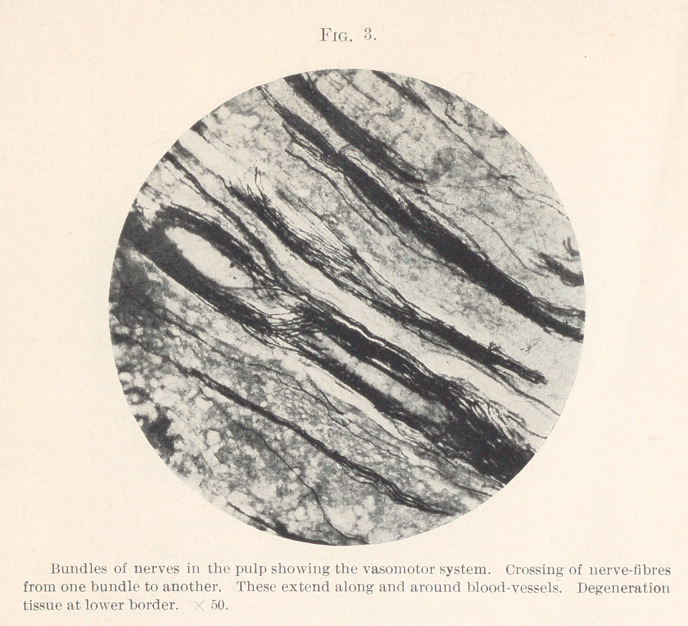


**Fig. 4. f4:**
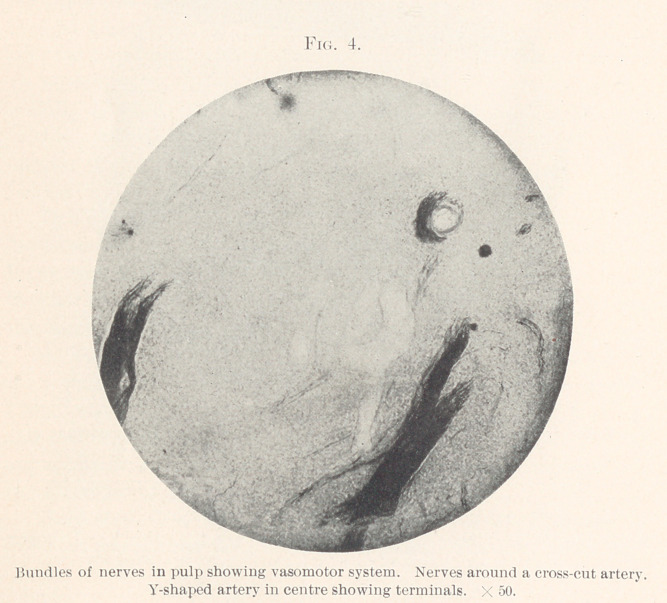


**Fig. 5. f5:**
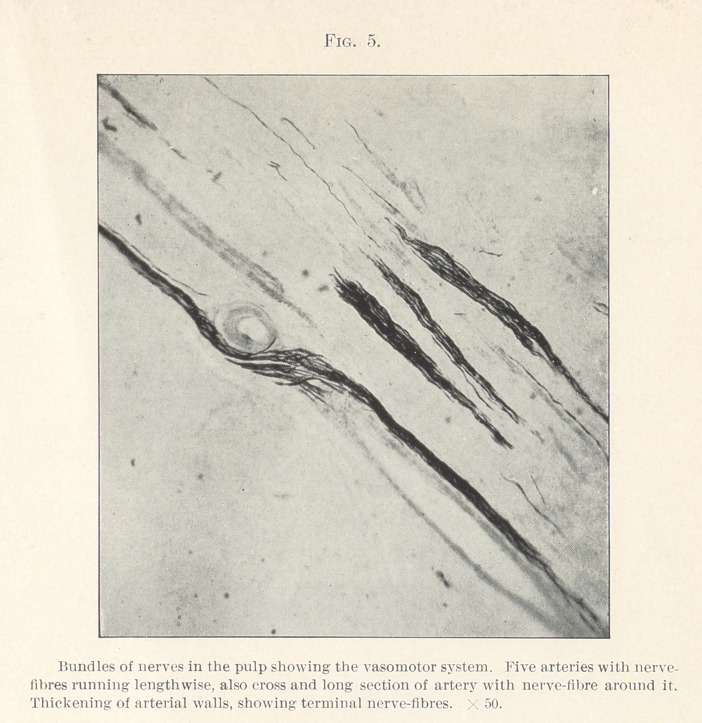


**Fig. 6. f6:**
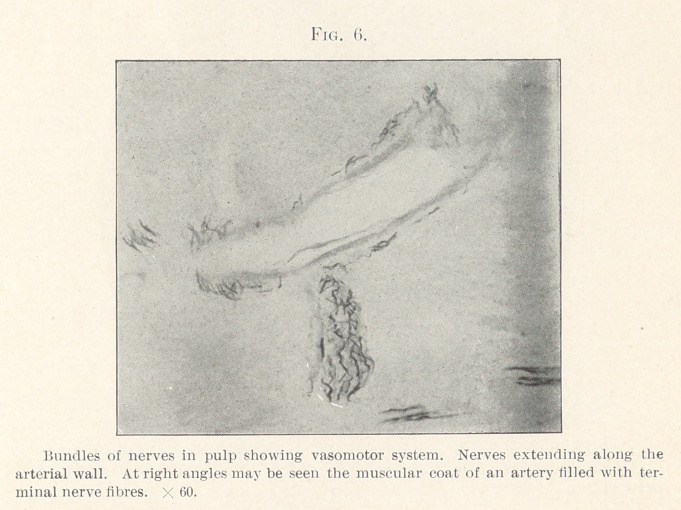


**Fig. 7. f7:**
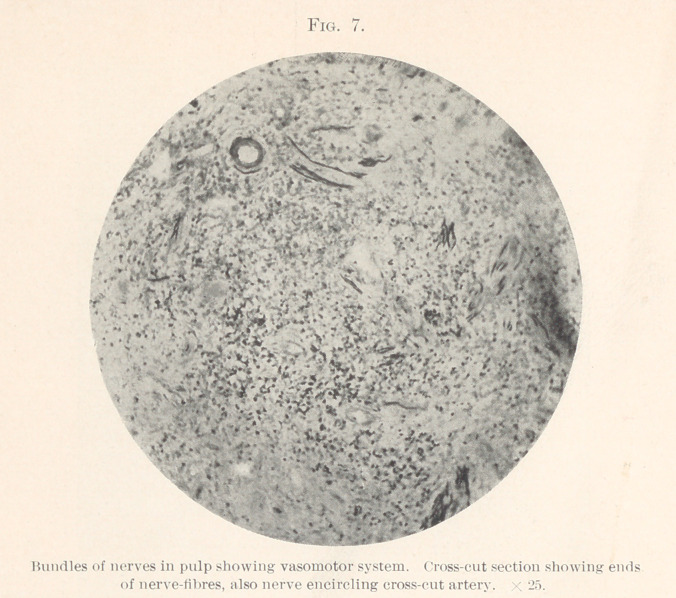


**Fig. 8. f8:**